# High molecular weight adiponectin as an adjunct biomarker for insulin dysregulation in equids

**DOI:** 10.1093/jvimsj/aalag186

**Published:** 2026-08-26

**Authors:** Melody A de Laat, Danielle M Fitzgerald, Fang I Li, Martin N Sillence, James M McGree

**Affiliations:** School of Biology and Environmental Science, Queensland University of Technology, Brisbane, Queensland, Australia; School of Agriculture and Food Sustainability, University of Queensland, Gatton, Queensland 4343, Australia; School of Biology and Environmental Science, Queensland University of Technology, Brisbane, Queensland, Australia; School of Biology and Environmental Science, Queensland University of Technology, Brisbane, Queensland, Australia; School of Mathematical Sciences, Queensland University of Technology, Brisbane, Queensland, Australia

**Keywords:** diagnostic test, equine metabolic syndrome, horse, hyperinsulinemia, laminitis, pony

## Abstract

**Background:**

Total adiponectin concentration is associated with hyperinsulinemia and laminitis risk in ponies but the high molecular weight (HMW) multimer is a more sensitive indicator of metabolic disease in other species and could be a useful biomarker for insulin dysregulation (ID).

**Hypothesis/Objectives:**

To determine a cut-off value for using HMW adiponectin as an adjunct diagnostic tool for ID.

**Animals:**

Cohort of 127 privately and university-owned horses and ponies of various breeds with 55 classified as ID positive and 72 as ID negative.

**Methods:**

Cohort study. Baseline and postprandial blood samples from an in-feed oral glucose test (0.75 g/kg dextrose) were used to determine ID status. Insulin and HMW adiponectin concentrations were measured using validated immunoassays. High molecular weight adiponectin cut-off values were derived with receiver-operator characteristic curve analysis using a bootstrap method to maximize the sum of sensitivity and specificity.

**Results:**

Median [IQR] HMW adiponectin concentration was higher (*P* < .001) in ID negative animals (4.19 [2-6.5] μg/mL) compared to ID positive animals (1.79 [0.63-3.5] μg/mL), with a cut-off value (95% CI) of 2.1 (1.26, 3.15) μg/mL (area under the curve 0.71; sensitivity 0.62; and specificity 0.74). Horses had higher (*P* < .001) HMW adiponectin concentrations (4.38 [2.22-7.3] μg/mL) than ponies (1.84 [0.64-3.75] μg/mL). However, HMW adiponectin did not differ between horses with and without ID, and both horse subgroups had concentrations higher (*P* ≤ .002) than ponies with ID.

**Conclusions and clinical importance:**

Use of a single cut-off value for HMW adiponectin as an adjunct biomarker for ID for both horses and ponies is not accurate.

## Introduction

Equine insulin dysregulation (ID) represents the single greatest risk factor for hyperinsulinemia-associated laminitis (HAL).^1–3^ Accurate diagnosis of ID is thus essential and the approach includes assessment of the animal’s history, clinical presentation, and multistep challenge test outcomes, such as insulin responsiveness to oral carbohydrate and insulin sensitivity.^4^ Functional assessments, such as the oral sugar test (OST) or oral glucose test (OGT) and insulin tolerance test, are current best practice for adequately understanding an individual’s degree of ID. As it is insulin that causes HAL, tests that directly assess insulin dynamics are always likely to be the most informative. However, testing for ID can be challenging and outcomes are not always definitive. The ability to use additional tests might improve outcomes and simplify monitoring of animals with disease. Thus, ancillary tests are being explored to support the primary evaluation tools for ID.^5^

Future ancillary tests for ID and equine metabolic syndrome (EMS) might include assessment of genetic markers,^6^^,^^7^ while current additional tests for EMS include measurement of total adiponectin, leptin, and triglycerides.^8^ These tests are used in research and clinically in some geographical locations to provide more information about the animal’s metabolic health and risk of HAL. Leptin can be used as an indicator of adiposity,^9^ while adiponectin has been shown to vary with ID status in some, but not all, studies^10^^,^^11^ and to indicate the risk of HAL^12^^,^^13^ in ponies. Given the direct association of hyperinsulinemia with laminitis, adiponectin could be a promising adjunct test for ID associated with EMS or pituitary pars intermedia dysfunction (PPID). However, the suitability of adiponectin as an adjunct biomarker for ID needs more consideration.

Adiponectin circulates in 3 forms of which the high molecular weight (HMW) multimer is the most biologically active in other species^14^ and the most sensitive marker of metabolic disease in humans.^15^ Low concentrations of HMW adiponectin are found in humans with metabolic dysfunction,^16^ horses and ponies with lower insulin sensitivity^17^ and with ID associated with EMS.^11^ However, studies to date have mostly measured total adiponectin and few studies have been undertaken in horses. Also, HMW adiponectin concentrations have not been compared between horses and ponies. This study aimed to determine whether a cut-off value could be derived for HMW adiponectin so that it can be used as an adjunct biomarker for ID.

## Materials and methods

This cohort study used samples from 127 horses and ponies, of which 36 were collected prospectively, and 91 were archived from previous studies.^18–21^ The criteria for inclusion were that each animal had received (1) a clinical examination including body condition score (BCS: 1-9)^22^ and cresty neck score (CNS: 0-5)^23^ and (2) an OGT. The exclusion criteria were animals aged < 3 years old, entire males, pregnant or lactating females, co-morbidities other than EMS and PPID, and being a donkey or mule. The in-feed OGT used a dextrose dose of 0.75 g/kg bodyweight (BW) delivered in 0.3% BW lucerne chaff, 100-200 g wheat bran and ~ 500 mL of water, with > 70% of the test meal consumed in all cases. The OGT was used to classify each animal as positive (insulin concentration ≥ 20μIU/mL at baseline and/or ≥ 85μIU/mL postprandially) or negative for ID.^1^ A diagnosis of PPID was made using seasonally adjusted, locally derived cut-off values for plasma adrenocorticotrophic hormone (ACTH) concentration and clinical examination findings.^24^ An analysis of the cohort without the PPID-affected animals was also performed for the cut-off derivations. A subset of 11 animals with a history of clinically recognized (≥2 episodes) HAL was examined for HMW adiponectin concentration. For comparisons between horses and ponies the animals were categorized by height (pony ≤ 144 cm).

Baseline (insulin and ACTH concentration) and postprandial (insulin only) blood samples were analyzed at a commercial veterinary diagnostics laboratory (Vetnostics, Perth, WA, Australia), while plasma HMW adiponectin concentrations in the baseline samples were measured in-house using a previously validated^25^^,^^26^ ELISA according to the kit protocol. When the concentration was below the limit of detection for an assay the detection limit value was used. Values that exceeded the upper limit were diluted and the assay repeated.

The data were analyzed for normality of distribution (Shapiro–Wilk test) which informed whether parametric or nonparametric tests were used. Comparative analyses used a *t-*test, rank-sum test, signed rank test, Spearman’s test, or ANOVA on ranks with Dunn’s coefficient. To determine an optimal value for cut-off a metric that summarized classification performance (eg, summation of sensitivity and specificity^27^) was used. A bootstrap approach, where the original dataset was repeatedly resampled, was used and for each resampled dataset the optimal cut-off value was determined.^28^ Repeating this process over a number of simulated datasets allowed bootstrap distributions to be generated for key diagnostic metrics, such as accuracy, sensitivity, and specificity. The bootstrap distributions approximate the sampling distribution of these summaries which allowed 95% CIs to be determined.

## Results

### Animals

This heterogeneous cohort of 47 mares and 80 geldings contained 55 horses and 72 ponies and comprised 21 different breeds (including hot, warm, and cold-blooded breed types, File S1). The mean ± SE age was 14.2 ± 0.53 years, and the mean ± SE BW was 362 ± 16.6 kg. Height at the wither ranged from 73.1 to 180 cm. Nine animals were positive for PPID and either previously diagnosed by the attending veterinarian (*n* = 6; all were managed with pergolide) or diagnosed during study testing (*n* = 3). The mean ± SE plasma ACTH concentration for the cohort was 27.6 ± 4.82 pg/mL. Based on the results of the OGT 72 animals were classified as negative for ID and 55 as ID positive. The OGT and morphometric data for these groups are provided in Table 1. There were 4 animals with PPID in the ID group, and 5 in the group without ID.

**Table 1:** The outcomes from diagnostic testing for ID reported as median [IQR] for 127 horses and ponies.

	**Negative for ID**	**Positive for ID**	***P* value**
***n***	72	55	–
**Sex**	28 F; 57 M	34 F; 36 M	–
**Type**	36 H; 26 P; 10 MH	19 H; 31 P; 5 MH	–
**Age (years)**	13.1 ± 0.67	15.6 ± 0.83	.02
**Bodyweight (kg)**	394 ± 23.3	320 ± 22.3	.04
**BCS (1-9)**	5 [5-6]	6 [5-6]	.17
**CNS (0-5)**	1 [1-2]	2 [2-3]	<.001
**0 h insulin (μIU/mL)**	2 [2-2]	12 [3-38]	<.001
**2 h insulin (μIU/mL)**	25 [14-45.8]	192 [111-280]	<.001
**ACTH (pg/mL)**	19 [14-27.5]	18 [12-25]	.3

Abbreviations: ACTH = adrenocorticotropic hormone; BCS = body condition score; CNS = cresty neck score; H = horse; ID = insulin dysregulation; MH = miniature horse; P = pony.

### HMW adiponectin and clinical examination findings

**Figure 1:**
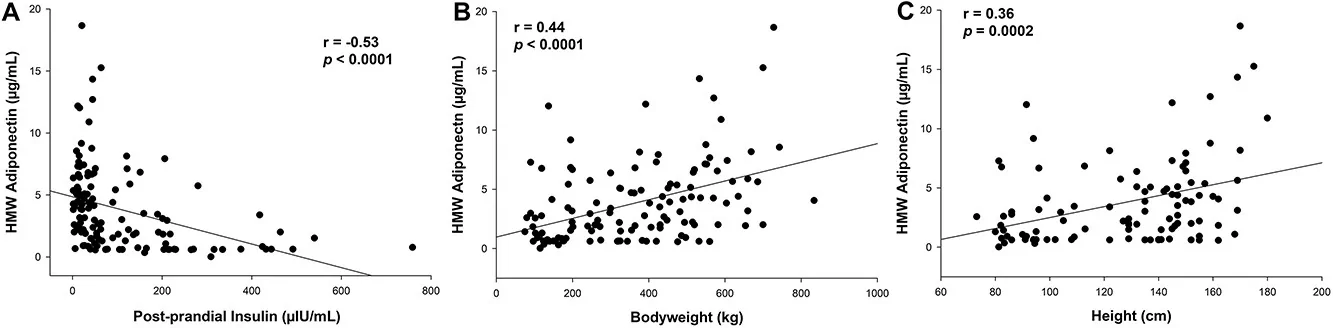
HMW adiponectin was negatively correlated with postprandial insulin concentration measured 2 h after an oral glucose test meal (panel A). Note that one outlier value was removed from the graph in panel A to enable better visualization of the data. It was also positively correlated with bodyweight (panel B) and height at the wither (panel C) in a cohort of 127 horses and ponies. Abbreviation: HMW = high molecular weight.

The median [IQR] HMW adiponectin concentration for the entire cohort was 2.76 [1.06-5.44] μg/mL. The HMW adiponectin concentration was not correlated with 0 h insulin concentration (*r* = −0.13; *P* = .16) but was negatively correlated (*r* = −0.53; *P* < .0001) with 2 h insulin concentration (Figure 1A). After visual inspection of the distribution of the data points a quadratic regression model was subsequently fitted to the data to further examine the nature of association between 2 h insulin and HMW adiponectin concentrations. Both linear (*P* = .0004) and quadratic (*P* = .049) terms were significant, confirming a modest curvilinear (U-shaped) association. However, the model explained a small proportion of the variance (*R*^2^ = 0.17; adjusted *R*^2^ = 0.15) in HMW adiponectin.

Plasma HMW adiponectin concentration was not correlated (*r* = 0.11; *P* = .24) with plasma ACTH concentration. The animals with clinically recognized, chronic HAL had a median HMW adiponectin concentration of 0.63 [0.6-0.7] μg/mL. The HMW adiponectin concentration did not differ (*P* = .29) between mares (2.97 [1.30-5.87] μg/mL) and geldings (2.52 [0.91-5.13] μg/mL) nor was it associated with age (*r* = −0.14, *P* = .12). The HMW adiponectin concentration was associated with BW (Figure 1B) and height (Figure 1C). There was no association between HMW adiponectin concentration and BCS (*r* = −0.11, *P* = .21) nor CNS (*r* = −0.13, *P* = .14).

### HMW adiponectin and insulin dysregulation

**Figure 2:**
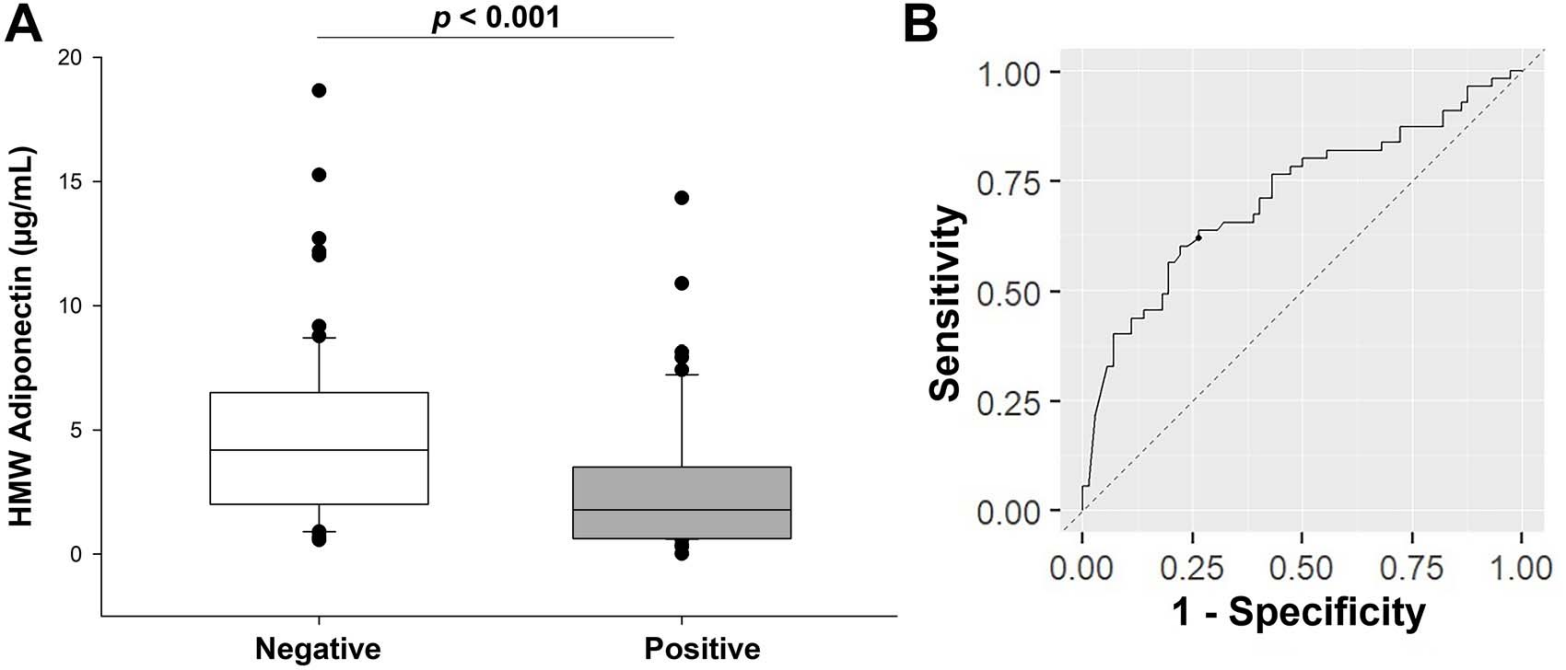
The median [IQR] plasma HMW adiponectin concentration (μg/mL) was higher in animals that were negative for ID, compared to animals positive for ID in a cohort of 127 horses and ponies (panel A). The receiver-operating characteristics curve for identification of a cut-off value for the diagnosis of ID had an area under the curve of 0.71 (panel B). The dashed line is the line of identity. Abbreviations: HMW = high molecular weight; ID = insulin dysregulation.

The HMW adiponectin concentration in the ID negative animals was higher (*P* < .001) than in the ID positive group (Figure 2A). For ID positive animals, the 0 h HMW adiponectin concentration was not correlated with the 0 h insulin concentration (*r* = 0.15; *P* = .28) but was negatively correlated with the 2 h insulin concentration (*r* = −0.52, *P* < .001). Similarly, for the animals that were negative for ID, the 0 h HMW adiponectin concentration was not correlated with the 0 h insulin concentration (*r* = 0.17; *P* = .16) but was negatively correlated with the 2 h insulin concentration (*r* = −0.33, *P* = .005).

Derivation of a decision limit for HMW adiponectin that distinguished between animals that did, and did not, have ID yielded a cut-off value of 2.1 μg/mL with a (bootstrap) 95% CI of (1.26, 3.15). The area under the curve (AUC) for this value was 0.71 (Figure 2B), suggesting a moderately accurate marker for disease.^29^ Sensitivity and specificity were 0.62 and 0.74, respectively. For this cohort, using this cut-off value would have resulted in a correct diagnosis (as designated by the gold standard method) 69% of the time and as such would have classified 87 animals correctly (34 true positives and 53 true negatives). Misclassification would have arisen for 40 animals with 21 false negatives and 19 false positives. When the animals with PPID were removed from the cohort and the cut-off analysis repeated, a cut-off value of 2.1 μg/mL with a (bootstrap) 95% CI of (1.44, 3.65) was derived. The AUC, sensitivity, and specificity were 0.68, 0.56, and 0.75, respectively.

### Comparison of horses and ponies

#### Insulin

One animal in the pony group was substantially more insulin dysregulated than the rest of the cohort (Table 2). However, while this outlier was removed from Figure 1A to facilitate visualization of the data, it was not removed from the dataset as nonparametric tests were used for analysis. The 0 and 2 h insulin concentrations differed (*P* < .001) for both the horse and pony subgroups without ID, as well as for ponies with ID. However, horses with ID had a less marked (*P* = .05) difference between the 0 and 2 h sampling timepoints (Table 2). Horses and ponies without ID also had a less marked (*P* = .05) difference in insulin concentrations at the 2 h timepoint (Table 2).

**Table 2:** Median [IQR] 0 h (baseline) and 2 h (postprandial) serum insulin concentrations (μIU/mL) for horse and pony subgroups with and without ID.

**Type**	**Sample size (*n*)**	**0 h insulin (μIU/mL)**	**2 h insulin (μIU/mL)**	***P* value**
**Negative for ID**				
** Horse**	36	2 [2-3.75]	19 [12-42.5]	<.001
** Pony**	36	2 [2-2]	34 [16.5-48]	<.001
*** P* value**		.62	.05	
**Positive for ID**				
** Horse**	19	29 [10-58]	122 [37-206]	.05
** Pony**	36	7 [2.25-34.3]	210 [143-361]	<.001
*** P* value**		.36	.12	

Abbreviation: ID = insulin dysregulation.

#### HMW adiponectin

**Figure 3:**
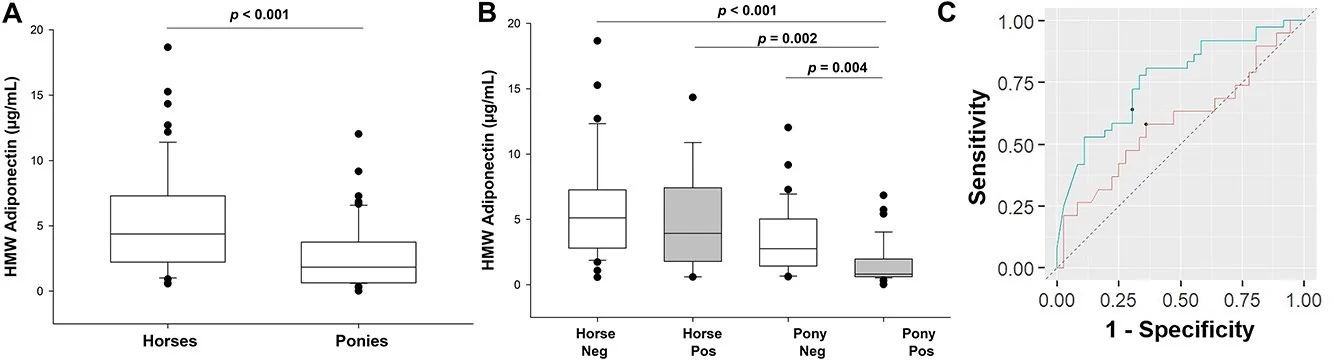
The median [IQR] plasma HMW adiponectin concentration (μg/mL) was higher in horses than ponies (panel A). Horses with and without ID and ponies without ID all had higher HMW adiponectin concentrations than ponies with ID (panel B). Two receiver-operating characteristics curves for identification of separate cut-off values for diagnosis of ID in horses (red line) and ponies (blue line) are shown in panel C. The dashed line is the line of identity. Abbreviations: HMW = high molecular weight; ID = insulin dysregulation.

The HMW adiponectin concentration was higher (*P* < .001) in horses than ponies (Figure 3A). However, the HMW adiponectin concentration did not differ between horses with (*n* = 19) and without (*n* = 36) ID (Figure 3B). Furthermore, horses had a higher (*P* ≤ .002) HMW adiponectin concentration than insulin-dysregulated ponies, whether they were positive for ID or not (Figure 3B). Subsequently, separate HMW adiponectin cut-off values were calculated for horses and ponies. The cut-off value that maximized the sum of sensitivity and specificity (4.16 μg/mL; Table 3) in horses had an accuracy of 62% (Figure 3C). Use of this cut-off would have classified 34 animals correctly (11 true positives and 23 true negatives). Misclassification would have occurred in 21 animals with 8 false negatives and 13 false positives. By comparison, a cut-off value of 1.57 μg/mL (Table 3) in ponies had an accuracy of 67% (Figure 3C), which would have classified 48 animals correctly (23 true positives and 25 true negatives). Misclassification of 24 animals would have occurred with 13 false negatives and 11 false positives. Similar cut-off values were derived for the horse and pony groups when this analysis was repeated with the animals with PPID removed from the cohort (Table 3).

**Table 3:** Cut-off values derived using ROC analysis for use of HMW adiponectin as a diagnostic biomarker for insulin dysregulation in horses and ponies. Analyses were performed with the full cohort, and with 9 animals with PPID removed.

**Type**	**Cut-off (95% CI)**	**AUC**	**Sensitivity**	**Specificity**
**Horse**	4.16 (1.85, 6.42)	0.59	0.58	0.64
**Pony**	1.57 (1.04, 2.49)	0.76	0.64	0.69
**Horse (no PPID)**	4.27 (2.82, 7.69)	0.54	0.53	0.58
**Pony (no PPID)**	1.85 (1.05, 2.7)	0.74	0.71	0.69

Abbreviations: AUC = area under the ROC curve; HMW = high molecular weight; PPID = pituitary pars intermedia dysfunction; ROC = receiver-operating characteristic.

## Discussion

This study investigated whether HMW adiponectin concentrations could be used to differentiate animals with and without ID and derived decision limits for its use as an adjunct biomarker for ID, and in doing so the aims were achieved. A key finding was that the HMW concentration of the horses was similar whether they had ID or not, while ponies with ID had lower HMW adiponectin concentrations than horses, and ponies without ID. As a result of this difference between groups, the cut-off value of 2.1 μg/mL for making a binary classification of ID positive/ID negative was not very sensitive, and 40 animals would have been misdiagnosed, with 21 false negatives. This finding is not inconsistent with a meta-analysis reporting that adiponectin (both total and HMW studies were included) was only a moderately accurate diagnostic biomarker for Metabolic Syndrome in humans.^30^ Due to the lack of data regarding an association between adiponectin and PPID, the cut-off values were also derived without PPID-affected animals included. However, their removal had little effect on the cut-off values in this study. Given the small number of animals with PPID in this study, separate studies that investigate the association between HMW adiponectin and animals with ID and PPID are warranted. The sensitivity of the cut-off is concerning as failure to recognize ID and any associated risk of laminitis could lead to inappropriate management strategies that fail to mitigate the unrecognized risk. Our data showing the marked difference in HMW adiponectin concentration between horses and ponies likely underpins the suboptimal performance of HMW adiponectin as a biomarker for ID in this cohort when attempting to use it as a single decision limit across all breeds.

Concentrations of HMW adiponectin in horses were > 2-fold higher than ponies, which was a novel and interesting outcome of the study, especially considering the large number of horse and pony breed represented in the cohort. Previous studies have often used more uniform cohorts comprising only one, or a few, breeds. One study that did compare horse (Standardbred, Andalusian) and pony (mixed-breed) groups found no difference in HMW adiponectin concentrations between breed groups and the range of concentrations reported was similar to those in the current study.^17^ Another study reported a median HMW concentration of 2.4 μg/mL for Welsh ponies with ID, compared to 8 μg/mL without ID.^11^ These values were slightly higher than those of our pony cohort that contained multiple breeds, including Welsh, Shetland, and various riding ponies. It is worth noting here that our findings relate only to the geographical location studied and the specific assay used. We also found an overall association with height, so larger cohort studies that compare specific breed groups might identify other differences among certain breeds for HMW adiponectin concentration.

Overall, the HMW adiponectin concentration was ~ 2.3-fold lower in animals with ID. We also found a negative correlation between postprandial insulin concentration and HMW adiponectin, which is consistent with the association with ID given that diagnosis is partly achieved using postprandial insulin values. These data are consistent with some previous reports in ponies where HMW adiponectin was inversely correlated with postprandial insulin concentrations during an oral glucose challenge test.^26^^,^^31^^,^^32^ However, another study found no association between HMW adiponectin and postprandial insulin in ponies.^33^ Studies have also found associations with basal insulin concentration,^25^ which we did not. Notably, differences between the time of sampling and fed state could explain the differences between studies. These data are all consistent with adiponectin being an insulin-sensitizing hormone in horses, as it is in humans.^34^ The relationship between HMW adiponectin and postprandial insulin concentration in this study was complex with a negative linear coefficient combined with a positive quadratic coefficient. This suggests a U-shaped relationship with an initial decrease in HMW adiponectin concentration with increasing postprandial insulin, that recovers somewhat at higher postprandial insulin concentrations. However, most of the variability in HMW adiponectin was not explained by the quadratic model making it more difficult to draw definitive conclusions about the full extent of the nature of the relationship between the 2 variables. Metabolic disease is complex and nonlinear relationships between markers, particularly in association with insulin effectiveness and resistance, are not uncommon,^35^^,^^36^ and have been demonstrated in horses.^37^ The role of insulin resistance was not investigated in the current study and more studies that investigate relationships between adiponectin and insulin sensitivity are needed.^17^

The lack of difference in HMW adiponectin concentration between horses with and without ID shows that using HMW adiponectin concentration as a biomarker for ID in the horses in this study was inappropriate. A value was derived for the sake of confirming this and showed that the predictive power was low, where the AUC of 0.59 was between 0.5 and 0.7 which suggests poor accuracy,^29^ with an AUC of 0.5 no better than random chance. The pony-specific cut-off values also had suboptimal sensitivity for a condition where false negatives should ideally be minimized. However, the need to separate the cohort into horse and pony subgroups was not foreseen so further work that focuses on examining decision limits for ponies is required. Furthermore, the sample size for the pony group was not large enough to enable us to apply different statistical methods that could have specifically focused on identifying a cut-off value for ponies that provided better sensitivity without negatively affecting specificity.^38^ Considering the study limitation relating to group sample size, the current data suggest that further evaluation of the utility of HMW adiponectin as an adjunct biomarker for ID in ponies is warranted. Additional diagnostic aids for ID would be useful given that some ancillary tests, such as basal insulin concentration, have poor sensitivity. Studies have determined the sensitivity of basal insulin as a diagnostic tool for insulin resistance with test sensitivities varying from 0% (compared to the FSIGTT^39^) to 85% (using an optimized cut-off value compared to a euglycemic, hyperinsulinemic clamp^37^). When basal insulin concentration was compared directly with an OST in a field study the sensitivity of the test varied depending on the cut-off value selected, with a sensitivity of 25% at a basal insulin threshold for ID of 20 μIU/mL.^40^

There was no association between HMW adiponectin concentration and BCS or CNS in this study and this differs to other reports where lower adiponectin concentration was associated with increased fat mass^25^^,^^41^ and CNS.^26^ However, studies have also reported either no clear association of total^42^ and HMW^43^ adiponectin with adiposity in ponies or a complex relationship where insulin sensitivity is more strongly associated with total adiponectin than body mass.^44^ A previous study of horses reported a cut-off value of 1.46 μg/mL for using HMW adiponectin to define an animal as lean or obese.^45^ However, the AUC used for derivation of this value was below the threshold of 0.5 so other markers needed to be combined with adiponectin for a satisfactory discriminatory outcome which underscores that adiponectin is not a good independent indicator of obesity. Collectively, these data suggest that the relationship between adiponectin and increased adiposity is independent of ID, and multiple diagnostic markers should be considered together. Of note, the range of adiposity scores in the current study was small, and this might have affected our ability to detect any correlation with adiposity here. The current consensus in the literature suggests that leptin, another adipokine, is a better measure of generalized adiposity, whereas adiponectin is more often associated with CNS and metabolic disease.^17^^,^^26^^,^^44^

The importance of understanding the relationship between adiponectin and ID is highlighted by the increasing number of studies that report a strong association between adiponectin concentration and laminitis risk. An early study showed that previously laminitic ponies had lower total adiponectin concentrations (2.1 μg/mL), than non-laminitic ponies (3.4 μg/mL).^46^ A large cohort study reporting total adiponectin concentrations derived a cut-off value of 2.5 μg/mL for laminitis development in ponies after 1 and 2 years of follow-up.^13^ In another prospective study a group of ponies that were classified as at high risk of developing laminitis had a median total adiponectin concentration of < 4.2 μg/mL.^12^ The very low HMW adiponectin concentration of the clinically recognized, chronically laminitic animals in the current study is not inconsistent with these values, despite the obvious problem of needing to try and compare HMW to total concentrations and the small sample size used to derive the value. It is worth noting that other animals in this study cohort could have had chronic HAL that was not clinically recognized, which is why the HMW adiponectin concentration of the laminitic animals was not directly compared with the rest of the cohort. It would be beneficial to derive a decision limit for laminitis risk for HMW adiponectin in future prospective studies.

There was no effect of sex or age on HMW adiponectin concentration, which is consistent with a previous study in horses,^25^ but differs to another study where total adiponectin concentration was lower, and baseline insulin concentration higher, in older horses.^47^ Data on the relationship between age and adiponectin from other species go beyond finding a simple linear relationship, where although concentrations are higher in older humans, it seems that high adiponectin concentrations are a predictor of good health in advanced age and longevity.^48^^,^^49^ The same has been reported for dogs where higher adiponectin correlated with reduced age-associated frailty.^50^ In horses there are no data on whether high adiponectin concentrations improve longevity, but an association with reduced laminitis risk suggests that this is a hypothesis worth investigating. Certainly, aging in horses has been associated with hyperinsulinemia and ID,^51–53^ which suggests that the relationship between adiponectin and age also needs to be examined with consideration of insulin status. The effect of sex on adiponectin is clearer in humans with females consistently reported to have higher adiponectin concentrations.^54^ Studies have shown that there is an effect of visceral fat and sex hormones on adiponectin, in particular a testosterone effect contributing to the lower adiponectin concentration in men,^54^^,^^55^ which obviously would not be detectable in equine studies that use geldings. Some studies have reported that female horses have a higher incidence of ID.^53^ Research that includes stallions is uncommon but would likely be required to identify a true sex effect here.

Overall, this study has provided data to suggest that use of a single cut-off for HMW adiponectin as an adjunct test for ID in both horses and ponies is not advisable, as horses have higher HMW adiponectin concentrations than ponies in both the non-insulin dysregulated and insulin dysregulated states. The cut-off value derived for the horses in this cohort was not an accurate indicator of ID and studies in larger cohorts of both horses and ponies are required to further investigate whether it might be useful as an adjunct biomarker for ID. Future work should focus on the investigation of cut-off values for HMW adiponectin in relation to laminitis risk.

## Supplementary Material

Supplementary_file_1_aalag186

## Off-label antimicrobial declaration

The authors declare no off-label use of antimicrobials.

## Institutional animal care and use committee or other approval declaration

The collection of new samples was approved by the Animal Ethics Committee of Queensland University of Technology (AE 2024-8480-18968) and procedures were carried out in accordance with relevant approvals and guidelines. All owners gave written consent for the use of their animal(s) for research purposes.

## Appendix

### Detailed materials and methods

#### Study design

This study used samples from 127 horses and ponies, of which 36 were collected prospectively, and 91 were archived from previous studies.^18–21^ All animals were located in South-East Queensland, Australia, and were either privately owned or part of a university research herd. All owners gave written consent for the use of their animal(s) for research purposes. The criteria for inclusion were that each animal had received (1) a clinical examination by the first author that included an assessment of body condition score (BCS; Henneke scale of 1-9)^22^ and cresty neck score (CNS; Carter scale of 0-5)^23^ and (2) an oral glucose test (OGT). The exclusion criteria were animals aged < 3 years old, entire males, pregnant or lactating females, co-morbidities other than equine metabolic syndrome and pituitary pars intermedia dysfunction (PPID) and being a donkey or mule.

The diagnostic test for insulin dysregulation (ID) was an in-feed OGT with a dextrose dose of 0.75 g/kg BW delivered in 0.3% BW lucerne chaff, 100-200 g wheat bran and ~ 500 mL of water. This test relied on voluntary consumption of the test meal and > 70% of the test meal was consumed in all cases. Tests were performed as close to 8 am as practicable in all cases and none of the animals had been fed a meal so were either fasted or had been allowed access to a small amount of pasture prior to the test. A weight tape was used to estimate the body weight of 11 animals due to logistical or behavioral issues. The remainder were weighed using an electronic scale (Active Scales, Victoria, Australia). The results of the OGT were used to classify each animal as positive (insulin concentration ≥ 20μIU/mL at baseline and/or ≥ 85μIU/mL postprandially) or negative for ID. The decision limits for a negative outcome were serum insulin concentrations of ≤19μIU/mL at 0 h as well as ≤84μIU/mL at 2 h.^1^ In borderline cases (*n* = 5) the BCS and CNS were also considered. A diagnosis of PPID was made using seasonally adjusted, locally derived cut-off values for plasma adrenocorticotropic hormone (ACTH) concentration and clinical examination findings.^24^ Within the ID positive group, a subset of 11 animals (1 horse; 10 ponies) with a known history of chronic (≥2 episodes), veterinary-diagnosed hyperinsulinemia-associated laminitis (HAL) was examined for median high molecular weight (HMW) adiponectin concentration. Diagnosis of HAL was made by each attending veterinarian using clinical examination and radiography. For comparisons between horses and ponies the animals were categorized by height, with animals ≤ 144 cm at the wither included in the pony group, which included miniature horses and miniature horse/pony crossbreds (*n* = 15).

#### Sample processing and assays

Blood samples were collected by jugular venipuncture before (within 30 min) and 2 h after provision of the OGT meal. The blood was immediately divided between clot activator and EDTA (prechilled) vacutainer tubes. Serum vacutainers were left at ambient temperature for 30-60 min then centrifuged at 1500×*g* for 15 min (Elmi CM-6MT, Newbury Park, CA, USA). The EDTA tubes were placed on ice for 10 min before centrifuging at 1500× *g* for 15 min. Plasma and serum fractions were separately aliquoted (1 mL) into 1.5 mL Eppendorf tubes and placed into a −20°C freezer until transfer to −80°C within 24 h. Archived samples had been collected in an identical manner. Serum insulin (Immulite 2000 XPi, Siemens, Forchheim, Germany) was measured in all samples and plasma ACTH concentration (Siemens Immulite 1000, Siemens, Forchheim, Germany) was measured in the 0 h samples by a commercial veterinary diagnostics laboratory (Vetnostics, Perth, WA, Australia). Plasma HMW adiponectin concentrations in the 0 h samples were measured using a previously validated^25^^,^^26^ ELISA (Cat. EZHMWAN-65 K, Merck Millipore, Darmstadt, Germany) according to the kit protocol. When the concentration was below the limit of detection for an assay the detection limit value was used. Values that exceeded the upper limit were further diluted with the kit sample dilution buffer and the assay repeated.

#### Statistical analyses

Statistical analyses were conducted using the R Project for statistical computing and SigmaPlot (v.15, Systat Software Inc, San Jose, CA, USA). The accepted significance level was *P* ≤ .05. Data were missing at random and therefore were ignored. Data are reported as mean ± SE or median [interquartile range]. The data were analyzed for normality of distribution (Shapiro–Wilk test) and analyzed with parametric tests if normally distributed or nonparametric tests if they did not follow a normal distribution or were ordinal (which included BCS and CNS). Comparative analyses used a *t-*test, rank-sum test, signed rank test, Spearman’s test, or ANOVA on ranks with Dunn’s coefficient for post-hoc analyses.

Using HMW adiponectin as a diagnostic tool for ID can be thought of as a binary classification problem where a cut-off value is used to indicate a positive or negative diagnosis. To determine an optimal value for this cut-off, a metric that summarizes classification performance (eg, summation of sensitivity and specificity) is ideal.^27^ In this study, we adopted a robust method based on a bootstrap resampling approach where the original dataset was repeatedly resampled with replacement to create multiple simulated datasets. For each resampled dataset an optimal cut-off value was determined.^28^ Repeating this process over a large number of resampled datasets allowed bootstrap distributions to be generated for key diagnostic metrics, such as accuracy, sensitivity, and specificity. These bootstrap distributions approximate the sampling distributions of the metrics (ie, how they would vary if the population was repeatedly sampled) thus allowing 95% CIs to be determined. Initially, we applied this approach to determine the optimal cut-off values for the cohort as a whole and establish overall diagnostic performance. The analysis was then repeated for the cohort with the animals with PPID removed and within subgroups (horses versus ponies) to evaluate whether optimal cut-offs and test performance differed between these populations.

## Abbreviations

ACTH: adrenocorticotrophic hormone
AUC: area under the curve
BCS: body condition score
BW: bodyweight
CNS: cresty neck score
HAL: hyperinsulinemia-associated laminitis
HMW: high molecular weight
ID: insulin dysregulation
OGT: oral glucose test
PPID: pituitary pars intermedia dysfunction
ROC: receiver-operator characteristic
